# The Enterics for Global Health (EFGH) *Shigella* Surveillance Study in Peru

**DOI:** 10.1093/ofid/ofad655

**Published:** 2024-03-25

**Authors:** Katia Manzanares Villanueva, Tackeshy Pinedo Vasquez, Pablo Peñataro Yori, Lucero Romaina Cacique, Paul F Garcia Bardales, Wagner V Shapiama Lopez, Fiorella Zegarra Paredes, Karin F Perez, Silvia Rengifo Pinedo, Hermann Silva Delgado, Thomas Flynn, Francesca Schiaffino, Josh M Colston, Maribel Paredes Paredes Olortegui, Margaret N Kosek

**Affiliations:** Asociación Benéfica Prisma, Unidad de Investigaciones Biomedicas, Iquitos, Loreto, Peru; Asociación Benéfica Prisma, Unidad de Investigaciones Biomedicas, Iquitos, Loreto, Peru; Division of Infectious Diseases and International Health, School of Medicine, University of Virginia, Charlottesville, Virginia, USA; Asociación Benéfica Prisma, Unidad de Investigaciones Biomedicas, Iquitos, Loreto, Peru; Asociación Benéfica Prisma, Unidad de Investigaciones Biomedicas, Iquitos, Loreto, Peru; Asociación Benéfica Prisma, Unidad de Investigaciones Biomedicas, Iquitos, Loreto, Peru; Asociación Benéfica Prisma, Unidad de Investigaciones Biomedicas, Iquitos, Loreto, Peru; Asociación Benéfica Prisma, Unidad de Investigaciones Biomedicas, Iquitos, Loreto, Peru; Asociación Benéfica Prisma, Unidad de Investigaciones Biomedicas, Iquitos, Loreto, Peru; Facultad de Medicina Humana, Universidad Nacional de la Amazonia Peruana, Iquitos, Loreto, Peru; Division of Infectious Diseases and International Health, School of Medicine, University of Virginia, Charlottesville, Virginia, USA; Division of Infectious Diseases and International Health, School of Medicine, University of Virginia, Charlottesville, Virginia, USA; Faculty of Veterinary Medicine, Universidad Peruana Cayetano Heredia, Lima, Peru; Division of Infectious Diseases and International Health, School of Medicine, University of Virginia, Charlottesville, Virginia, USA; Asociación Benéfica Prisma, Unidad de Investigaciones Biomedicas, Iquitos, Loreto, Peru; Division of Infectious Diseases and International Health, School of Medicine, University of Virginia, Charlottesville, Virginia, USA

**Keywords:** Amazon, diarrhea, Loreto, *Shigella*, shigellosis

## Abstract

**Background:**

The Enterics for Global Health (EFGH) Peru site will enroll subjects in a periurban area of the low Amazon rainforest. The political department of Loreto lags behind most of Peru in access to improved sources of water and sanitation, per capita income, children born <2.5 kg, and infant and child mortality. Chronic undernutrition as manifested by linear growth shortfalls is common, but wasting and acute malnutrition are not.

**Methods:**

The recruitment of children seeking care for acute diarrheal disease takes place at a geographic cluster of government-based primary care centers in an area where most residents are beneficiaries of free primary healthcare.

**Results:**

Rates of diarrheal disease, dysentery, and *Shigella* are known to be high in the region, with some of the highest rates of disease documented in the literature and little evidence in improvement over the last 2 decades. This study will update estimates of shigellosis by measuring the prevalence of *Shigella* by polymerase chain reaction and culture in children seeking care and deriving population-based estimates by measuring healthcare seeking at the community level.

**Conclusions:**

Immunization has been offered universally against rotavirus in the region since 2009, and in a context where adequate water and sanitation are unlikely to obtain high standards in the near future, control of principal enteropathogens through immunization may be the most feasible way to decrease the high burden of disease in the area in the near future.

## Teaser

Loreto, Peru, has a high burden of shigellosis, diarrhea, and dysentery. The population is an appropriate one in which to study improved treatments or prevention strategies.

The enrollment for the Enterics for Global Health (EFGH) *Shigella* surveillance study in Peru is being carried out in the city of Iquitos, a metropolitan area of just under half a million inhabitants and capital of Loreto, the largest and most geographically isolated of Peru's 25 political departments. Iquitos is the main urban settlement in the otherwise sparsely populated Peruvian Amazon, the country's vast, flat, neotropical interior east of the Andes. Lacking connection to Peru's main road network, Iquitos is accessible only by air or by river transport (notably the largest city in the world for which that is the case) and ferry and boat transportation routes along the region's sprawling system of Amazon tributary waterways connect the urban population of Iquitos with the many smaller riverine communities of Loreto and with Ecuador, Colombia, and Brazil. Located just 400 km south of the equator, Iquitos’ tropical rainforest climate [1] makes it warm and humid year-round, with total annual rainfall of 2.8–3.0 m and seasons defined principally by river levels. Heavy rains occur year-round but are most intense between December and March and accompanied by rising river waters and flooding that peak in early April. Rainfall and river levels subside to a minimal level in late August before increasing slowly. This low-lying area (just 114 m above sea level) is highly vulnerable to extreme rainfall events, notably the 2011–2012 La Niña–related flood, which lasted for 6 months and caused increased transmission of numerous enteropathogens including *Shigella* [2], as well as widespread population displacement and crop failures [3]. Such events are becoming more frequent and extreme as a result of climate change [4].

**Figure 1.**
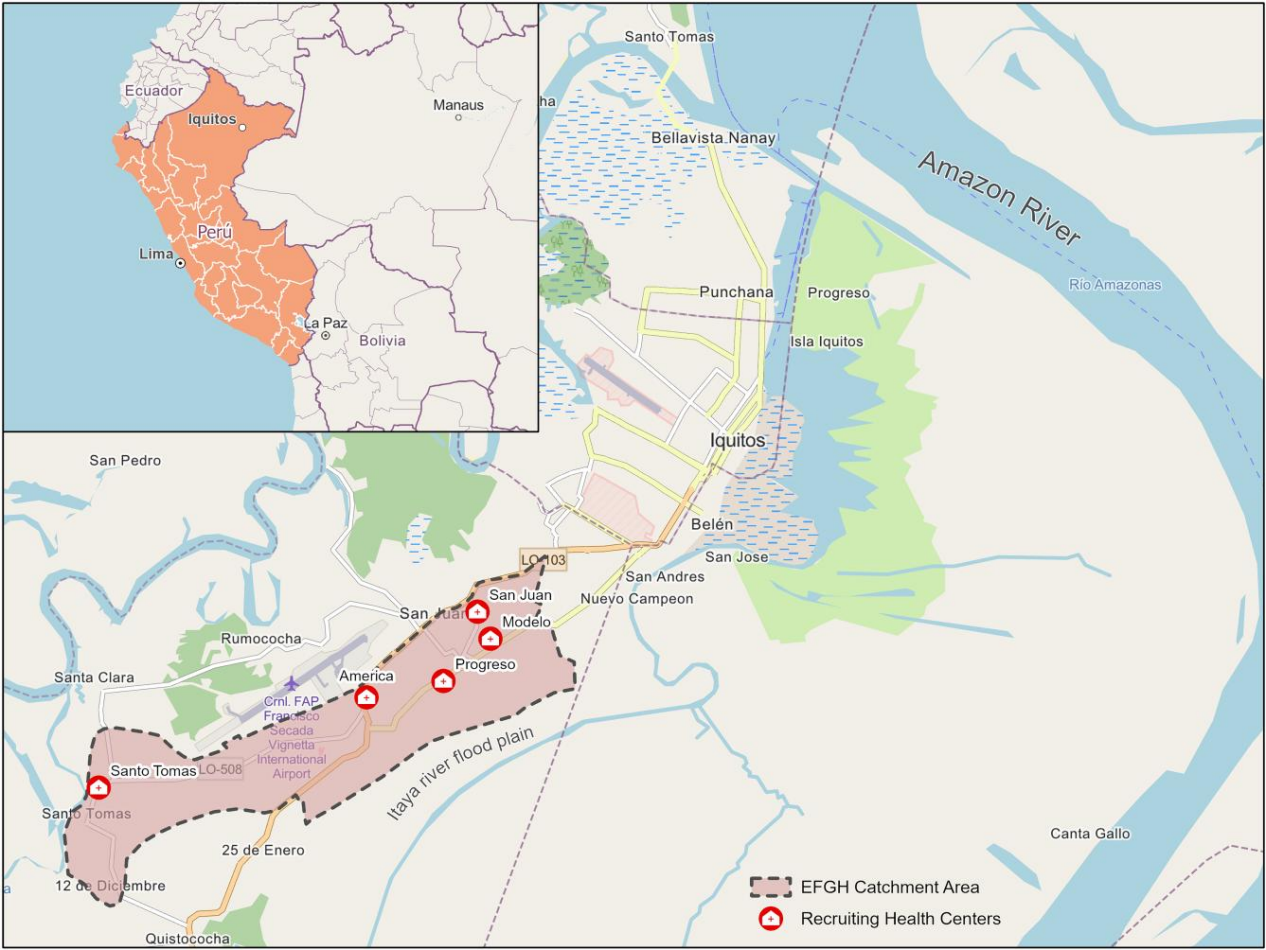
Catchment area and locations of recruiting health facilities in the Enterics for Global Health (EFGH) study site in Iquitos, Peru. Base and inset map data ©OpenStreetMap contributors, Microsoft, Facebook Inc and its affiliates, Esri Community Maps contributors, map layer by Esri, modified by authors. Health facility data provided by Instituciones Prestadores de Servicios de Salud, Maynas, Loreto.

The greater Iquitos metropolitan area is divided into 4 districts (the smallest administrative level of Peru): Iquitos, Belen, Punchana, and San Juan Bautista. The EFGH site itself is contained within the largely periurban San Juan Bautista district in the city's southwest and comprises the contiguous catchment areas of 5 primary healthcare facilities, bounded on the northwest by the airport and major thoroughfare of Avenida José Abelardo Quiñones, and by the flood plain of the river Itaya to the southeast (Figure 1).

## DEMOGRAPHIC AND SOCIOECONOMIC CHARACTERISTICS

The Loreto region had around 883 500 inhabitants at the last census in 2017, with more than 493 000 living in the province of Maynas and 369 000 in the city of Iquitos [5]. Fertility rates in the region are the highest in the country by some margin (4.3 births per woman aged 15–49 years, compared to 2.3 nationally in 2012), reflecting widespread unmet need for family planning and cultural preferences for larger family sizes [6], and contributing to a population age structure that skews toward younger ages [7]. Thirty-seven percent of the population is between 0 and 14 years of age, 56.9% between 15 and 64 years, and only 5.9% ≥65 years [5]. The infant mortality rate in the region is approximately 30 deaths per 1000 live births, while in Lima, it reaches the lowest level in the country with 11 deaths per 1000 live births [8]. The childhood mortality in Loreto is 40 per 1000 live births, the highest in Peru.

Table 1 summarizes other key socioeconomic and child health indicators for Loreto and for Peru as a whole. In addition, the population of Iquitos has an illiteracy rate of 4.1% and the 2020 National Survey of Households found that 34.6% of the population in the Loreto region lives in poverty, while 39.3% is vulnerable to poverty and extreme poverty affects 8.1% of the population. Only 4% of the population of the province of Maynas self-identifies as indigenous.

**Table 1.** Socioeconomic and Child Health Indicators for the Region of Loreto Compared With National Rates for Peru [9]

Indicator	Regional (Loreto)	National (Peru)
Household characteristics		
Electricity	79.6%	96.1%
Public network water source	27.5%	79.1%
Sanitation connected to public sewage system	42.5%	70.6%
Household crowding^a^	32.1%	21.3%
Education		
Women completing primary education or higher	62.1%	69.7%
Men completing primary education or higher	65.2%	74.0%
Child health and nutrition		
Newborns with weight <2.5 kg	8.4%	6.7%
Children with stunting	23.6%	11.5%

^a^Three or more household members per room for sleeping.

## WATER AND SANITATION

Due to the prevailing socioeconomic, political, and environmental conditions, the provision of safe drinking water and adequate sanitation to the population remains an unmet basic need. According to the most recent national survey, only 27.5% of households in Loreto obtain drinking water from the public network, and 42.5% have sanitation facilities connected to the public sewerage system [9]. The city of Iquitos has a single potable water treatment plant located in the Pampachica neighborhood, which is fed by water from the Nanay River. However, the supply of this service is limited and intermittent with water pumped through the network 3 times per day (5–7 Am, 10 Am to 12 Pm, and 4–6 Pm), a schedule that has remained unchanged for the last 20 years. Moreover, coverage of this water network in its catchment population is incomplete, leaving a significant portion of the population to resort to obtaining water from wells or rivers. The entire city of Iquitos is served by a single wastewater treatment plant fed by underground pipes that has failed to operate since its construction in 2014, instead discharging sewage into populated areas untreated. Basic, unimproved latrines commonly discharge wastewater into open canals, water bodies, or vegetation areas, mostly without any physical barrier as heavy rainfall leads to the admixture of surface waters, which are near the majority of the households in the catchment area.

## HEALTH AND NUTRITION CHARACTERISTICS

### Nutrition and Food Security

The typical diet among children living in urban and periurban Iquitos is based around rice, grains, potatoes, and plantains. The most commonly consumed animal-source proteins are eggs, dairy, and poultry, followed by fish and pork [10, 11]. Due to Loreto's limited agricultural production, food availability, distribution, and diversity are strongly sensitive to climate, both environmental and political [10]. Coping strategies during times of food scarcity include informal food-sharing practices. As a result, older, larger, and more connected communities have been shown to be at lower risk of food insecurity [12, 13]. Extended networks between urban and rural communities are also associated with lowered risk of food insecurity during times of stress [12]. Population nutritional status in Loreto lags behind that of the rest of Peru. In Loreto, 23.6% of children are stunted by 2 years of age and 8.4% of newborns weigh <2.5 kg at birth [9].

### Infant Feeding

Breastfeeding practices have been well characterized in this population. Analyses of infant feeding data from the Etiology, Risk Factors and Interactions of Enteric Infections and Malnutrition and the Consequences for Child Health and Development (MAL-ED) cohort in the Santa Clara de Nanay suburb found a median duration of exclusive breastfeeding of just 19 days [11], but that predominant breastfeeding up to 6 months of age was common (around 50%) and that the average age at which infants transitioned from full breastfeeding was 172 days [14]. Water tends to be the first non–breast milk liquid consumed, followed by tea/coffee or semisolids like porridge or banana. Formula and animal milk use is relatively uncommon. Maternal age, parity, monthly per capita income, and maternal marital status were not associated with the age at which semi-solids were introduced, and maternal depressive symptoms at 6 months was the only maternal factor associated with the transition to partial breastfeeding [14].

### Vaccination

National vaccination coverage estimates in Peru are one of the highest in the region with over 70% to 80% coverage in children <15 months of age for vaccines included in the general vaccination program. Vaccines include BCG; poliomyelitis (inactivated polio vaccine for the first 2 doses and oral polio vaccine for the third dose); rotavirus; anti-pneumococcal; measles-mumps-rubella *Haemophilus influenzae* type b, diphtheria-tetanus-pertussis, and hepatitis B (pentavalent vaccine); and influenza. Regional estimates indicate that 60% or less of children <15 months of age have completed the basic immunization program [15]. Pediatric coronavirus disease 2019 (COVID-19) vaccines for children 5–11 years old were introduced in September 2022 (Table 2). As of May 2023, 42.6% of children in the country had received 2 doses of the vaccine, while 38% of children in Loreto had completed a 2-dose scheme [18]. Recently, 2 cases of acute flaccid polio were reported in Peru, serving as evidence that the disruption in healthcare services that occurred in remote areas of Peru greatly affected previously high rates of early childhood immunization coverage, brought on in part by decreased intensity of routine care during the COVID-19 pandemic [19].

**Table 2.** Immunization Schedule for Children <10 Years of Age in Peru

Vaccine	Schedule
BCG	Birth
Recombinant HepB	Birth
Pentavalent (DPT-HepB-Hib)	2 mo, 4 mo, 6 mo
IPV	2 mo, 4 mo
OPV	6 mo, 18 mo, 4 y
Rotavirus (Rotarix)	2 mo, 4 mo
Pneumococcal (13-valent)	2 mo, 4 mo, 12 mo
MMR	12 mo, 18 mo
Varicella	12 mo–3 y (1 dose)
Yellow fever	15 mo (1 dose)
Hepatitis A	15 mo (1 dose)
DPT	18 mo, 4 y
HPV	9 y
Influenza	<1 y; 2 doses separated by a month
	>1 y; 1 dose annually
COVID-19	Between 6 mo and 4 y: 2 doses separated by 28 d.
	Between 5 and 11 y: 2 doses separated by 28 d.

Source: Ministry of Health, Peru [16, 17].

Abbreviations: COVID-19, coronavirus disease 2019; DPT, diphtheria-pertussis-tetanus; HepB, hepatitis B; Hib, *Haemophilus influenza* type b; HPV, human papillomavirus; IPV, inactivated polio vaccine; MMR, measles-mumps-rubella; OPV, oral polio vaccine.

### Communicable Diseases

Loreto reports one of the highest rates of malaria, dengue, and acute diarrheal disease in the country. The region accounted for 83% of national malaria cases in 2021, an incidence of 1741 cases per 100 000 people, while the equivalent rate for dengue was 278 cases compared with 2.2 cases in Lima [20]. Ongoing surveillance of the etiology of acute febrile illness (AFI) in the greater Iquitos area has been established through RIVERA, a health facility–based case-control study implemented through a partnership between Asociación Benéfica Prisma, the University of Virginia, and the US Centers for Disease Control and Prevention [21]. Preliminary findings indicate that 12.4% of AFI cases are attributable to the dengue viruses, 8.2% to *Plasmodium* spp, and 5.2% to severe acute respiratory syndrome coronavirus 2 (SARS-CoV-2) (unpublished data). Peru was hit harder by the COVID-19 pandemic than many other comparable countries and experienced the highest national mortality rate for COVID-19 in the world at 666 deaths per 100 000 [22]. Within the country, Loreto was one of the first and most severely affected regions, such that by July of 2020, seroprevalence of SARS-CoV-2 antibodies in Loreto was estimated to have already reached 70% [23].

## OVERVIEW OF RECRUITMENT FACILITIES

The local healthcare system is split between the public and private sectors, and those with different insurance receive nonemergent care in different healthcare facilities. The public healthcare system is composed of 3 systems: (1) a public safety net plan (Seguro Integral de Salud [SIS]); (2) a workers’ social health insurance (EsSalud); and (3) a health insurance system exclusively available to the police and military forces. The largest provider plan in Loreto is the SIS, which covers vulnerable populations and provides universal primary care to children. It is entirely subsidized by the Peruvian government and overseen by the Ministry of Health of Peru. All EFGH recruitment centers are SIS-based care facilities. Primary healthcare facilities have 4 levels ranked based on the size of the population they serve and the level and complexity of care provided: levels I-1 and I-2 are referred to as health posts, while I-3 and 1-4 are health centers. Hospitals can be levels II-1, II-2, or III-3.

Health networks are comprised of a number of micro-networks, which themselves contain a specific number of health establishments of various categories. Loreto holds 8 health networks and 35 micro-networks with a total of 341 health establishments and 13 hospitals. The province of Maynas, where the EFGH study takes place, holds 1 health network and 4 micro-networks, and recruitment of subjects into the study will take place at 5 of the 16 health establishments of the Southern Iquitos micro-network [24]—C. S. San Juan Bautista (category I-4), C. S. America (category I-3), P. S. Santo Tomas (category I-2), P. S. Modelo (category I-2), and P. S. Progreso (category I-2) (Figure 1). These health establishments have basic services such as electricity (although power outages are common) and potable water. Service hours are limited, ranging from 7 Am to 1 Pm, and staff is generally composed by general practitioners, nurses, and technicians. Category I-1 to I-3 facilities refer patients to either I-4 establishments or hospitals, depending on the severity and complexity of the cases.

There are additional clinics near the catchment area that provide care to individuals with workers insurance (EsSalud) or insurance plans that are either linked to the military or police, or private for-profit. Private clinics are not a common source of medical care, although pharmacies may act to recommend therapies and treatment [25].

## SUMMARY OF DIARRHEA MANAGEMENT GUIDELINES

The management of acute watery diarrhea in Peru is based on national guidelines from 2017, and emergency management was further updated in 2022 [26, 27]. Hydration status is assessed according to the World Health Organization methodology to classify children with none, some, or severe dehydration with treatment plans A, B, and C, respectively, to tailor interventions to the degree of dehydration present [28]. Plan A in Peru recommends the use of low osmolarity oral rehydration solution. Plan B treatment uses the same treatment but recommends an initial in-clinic visit for a 4-hour observation period to delineate clinical response to treatment. Treatment plan C recommends immediate treatment with intravenous fluids or nasogastric fluids if the child is unable to drink or has repetitive vomiting or if supplies for intravenous therapy are not available. Oral therapy is to be given to conscious patients if they can drink in addition to intravenous fluids. In cases of severe dehydration and acute watery diarrhea, patients should receive 30 mL/kg in 30 minutes followed by 70 mL/kg over the next 2.5 hours. Patients are then reassessed after 3 hours of therapy and restaged for the degree of dehydration. In cases where intravenous rehydration is indicated, a polyelectrolyte formulation is preferred to normal saline [29]. Ringer's lactate is not favored based on relative cost considerations [26]. The routine use of antibiotics in the management of acute diarrhea is discouraged since the majority of cases of diarrhea are either viral or self-limited. Therapy with 20 mg a day of elemental zinc is recommended in all children between 6 months and 5 years of age for a period of 10 days [26]. Additional antidiarrheal agents are not recommended and the use of antiemetics, including ondansetron, is discouraged [26]. Restrictive diets and/or specialized formulas are to be avoided and nourishment with age and regionally appropriate diets, particularly breastfeeding, actively encouraged [26].

Epidemiologic definitions of diarrhea specify that the maternal report of blood in stool in a child with 3 or more liquid stools in a 24-hour period is to be considered dysentery. Clinical guidelines additionally identify the presence of macroscopic blood in stool and temperature of 39°C as signs of invasive diarrhea [26, 30]. Three antibiotics are recommended in the national guidelines for the management of dysentery: trimethoprim-sulfamethoxazole (5 mg trimethoprim component/kg per dose, every 12 hours), furazolidone (2 mg/kg/dose, every 6 hours), and nalidixic acid (10 mg/kg/dose, every 6 hours) for a 5-day period [31]. Follow-up should be planned at 2 days following the initiation of treatment; failure of clinical response at that time or clinical progression should lead to a stool culture and change in antibiotic. Despite this being the formal national recommendation, expert national practitioners have advocated for alternative management based on available evidence of resistance to recommended first-line therapies of ciprofloxacin (10–15 mg/kg/dose every 12 hours for 5 days) and azithromycin (10 mg/kg/day on day 1 followed by 5 mg/kg/day on days 2–5) as first- and second-line oral options with ceftriaxone (50–75 mg/kg/day) indicated in cases where parenteral therapy is indicated [32]. This is challenged by the fact that public health establishments will only be reimbursed for pharmaceuticals that are specified under the national guidelines. As a result, physicians associated with public health centers continue to prescribe trimethoprim-sulfamethoxazole and furazolidone as first treatment options for dysentery in many cases.

Illnesses presenting with multiple symptoms present an additional challenge to diarrhea management. According to World Health Organization guidelines, children presenting to health establishments will be diagnosed based on the principal symptom associated with mortality. As a result, multiple symptoms are generally reduced to a single illness entity. For instance, a child presenting with an acute lower respiratory infection (ALRI) in addition to diarrhea will be receive a diagnosis of ALRI, while mention of diarrhea or dysentery will not be registered in the health center logs.

## RECENT HISTORICAL PREVALENCE, CARE-SEEKING, AND MANAGEMENT OF DIARRHEA IN CHILDREN

**Figure 2.**
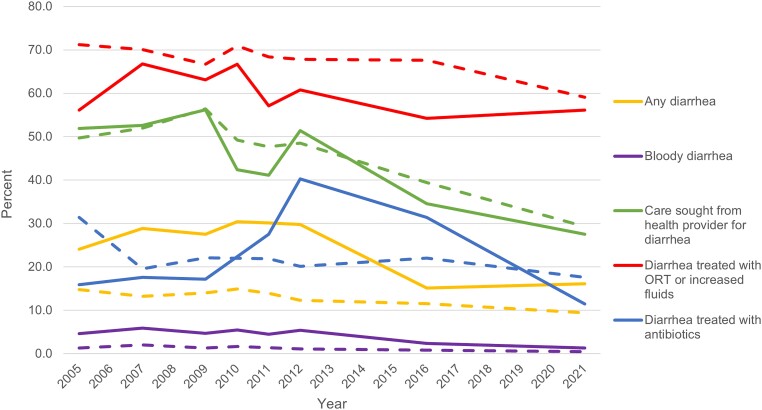
Trends in key indicators of burden, care-seeking, and management of diarrhea in children since 2005 for the Loreto region (solid lines) compared to all of Peru (dashed lines) [6, 8, 9]. Abbreviation: ORT, oral rehydration therapy.

Indicators of prevalence, care-seeking, and management of diarrhea in children are available at the national and regional levels from the Peru continuous Demographic and Health Surveys (cDHS) biannually and later annually from 2004 to 2014, and, since 2016, the 5-yearly demographic and family health surveys (ENDES) conducted by the Peruvian National Institute of Statistics and Information (see Figure 2) [6, 8, 9]. The 2-week period prevalence of diarrhea in children under 5 in Loreto as measured by the cDHS was consistently around double that of Peru as a whole and approached 30%. The 2 rounds of ENDES, however, estimated a regional prevalence that was almost half that (15.1% in 2016 and 16.1% in 2021), whereas national prevalence in ENDES was more consistent with the earlier cDHS estimates (trends that were mirrored in the prevalence of bloody diarrhea). The proportion of diarrhea cases in Loreto for which care was sought from a health provider declined from 56.2% in 2009 to 27.5% in 2021, similar to national levels. The proportion of diarrhea cases in Loreto that were treated with oral rehydration therapy or increased fluids fluctuated between 54.2% and 66.8% since 2005, consistently lower than the national rate, while the proportion treated with antibiotics peaked sharply in 2012 before declining to its lowest level of 11.5% in 2021.

## HISTORICAL *SHIGELLA* INCIDENCE, PREVALENCE, AND ANTIMICROBIAL RESISTANCE DATA

The history of shigellosis in Peru in many ways reflects broader global trends in *Shigella* epidemiology. Shortly after the initial report of shigellosis in Peru in 1942, culture-based methods found that 10.2% of samples from individuals with diarrhea in private clinics in the capital city of Lima from 1951 to 1957 were positive for *Shigella* [33]. Even in this early era, shifts in the proportion of disease caused by each species were apparent, with *Shigella flexneri* responsible for 49.9% of cases in 1951, which had increased to 76.2% just 6 years later [33]. *Shigella flexneri*, particularly serotype 2a, has remained the more prominent cause of shigellosis country-wide [34–37]. However, echoing global trends tied to urbanization and improved sanitation, *Shigella sonnei* has become more prevalent in recent years in more urban settings [36, 38–40]. Meanwhile, infection due to *Shigella dysenteriae* in Lima has declined over time, from almost 20% of shigellosis cases in 1953 [33], to 9% in 1985 [34], to 5% from 2008 to 2011 [37, 38], and absent from a group of 85 *Shigella* isolates tested in 2013 [36]. In Loreto between 2002 and 2006, 2.4% of the sampled pediatric cases were positive for *S dysenteriae* [41].

Peru has been no exception to the rule of worldwide emergence of antimicrobial resistance in *Shigella*. Resistance to chloramphenicol, the initial drug of choice [33], was observed in Peru as early as 1970 [42]. Modern studies of isolates sampled from Lima [35–37, 42, 43], Loreto [41, 44], and country-wide [38, 45] have shown the progressive development of resistance for trimethoprim-sulfamethoxazole (now consistently >75%) and nalidixic acid (5%–14%) [41–45]. Fluoroquinolone resistance was described in Lima as early as 1991 [42], though multiple subsequent studies have found no evidence of fluoroquinolone resistance, neither in Lima [36, 37] nor in samples from regional reference laboratories [45]. In Loreto, however, 3%–4% of isolates from 2002–2006 had intermediate or high resistance to ciprofloxacin [41]. Surveillance in Loreto was also responsible for identifying azithromycin resistance among 2%–5% of *Shigella* isolates [41, 44], and the region appears unique in Latin America for reporting circulation of both azithromycin- and fluoroquinolone-resistant phenotypes, albeit at a prevalence that still allows for their empiric clinical use [46].

While highly resistant isolates of *S sonnei* are well described in the literature, Peru reported its first 2 cases of infection due to *S flexneri* expressing the CTX-M–type extended-spectrum β-lactamase in 2013 [47]. These seemingly inevitable increases in resistance to multiple classes of antimicrobials, coupled with the fact that children aged <5 years in Loreto spend >4% of their lives taking antibiotics, a quarter of which are prescribed for diarrheal illness [25], underscore the need for vaccination as a means of addressing the burden of shigellosis in the Peruvian Amazon, as well as improved practices in antibiotic use among local health practitioners [25].

Two major community-based, multiyear cohort studies of diarrheal disease etiology have been carried out in southwest Iquitos and have demonstrated that the area has among the highest incidence rates of *Shigella* infection reported in the literature [41, 48]. The first took place from October 2002 to April 2006 and followed a cohort of 442 subjects aged <6 years through a combined 914.4 person-years of surveillance. *Shigella* was primarily detected by culture, with end-point polymerase chain reaction (PCR) only carried out to quantify underestimation of attribution in cases of clinical dysentery. Results presented in Table 3 are based on culture-based surveillance and demonstrate some of the highest rates of shigellosis recorded from longitudinal surveillance in the absence of an outbreak in the last 25 years. When PCR was conducted on specimens from episodes of dysentery, 64% were positive, although only 34.8% of dysenteric episodes were positive by culture, so rates reported are conservative. Speciation was performed on 404 isolates and serotyping was done on the 278 *S flexneri* isolates, which made up 68.8% of all *Shigella* infections. The most common serotypes were 2a (33.1%), 3a (19.4%), and 6 (16.5%).

**Table 3.** Age-Specific Incidence Rates per Child-Year of Diarrhea, Dysentery, and Diarrheal Episodes in Which the Stool Sample Tested Positive for *Shigella* spp and for *Shigella flexneri* Isolated by Culture

Age, mo	Diarrhea, Any Etiology	Dysentery, Any Etiology	*Shigella* spp–Positive Diarrhea	*Shigella flexneri*–Positive Diarrhea	Child-Years
0–5	6.84 (6.07–7.43)	0.06 (.02–.19)	0.23 (.13–.46)	0.17 (.08–.37)	47.1
6–11	7.21 (6.55–7.74)	0.19 (.10–.36)	0.16 (.08–.31)	0.08 (.03–.22)	62.4
12–23	7.29 (6.77–7.66)	0.36 (.27–.47)	0.43 (.34–.53)	0.30 (.23–.39)	150.3
24–35	4.99 (4.57–5.45)	0.34 (.25–.45)	0.40 (.31–.51)	0.27 (.20–.35)	161.3
36–47	3.52 (3.16–3.97)	0.16 (.11–.23)	0.39 (.30–.51)	0.26 (.19–.36)	165.4
48–59	2.59 (2.30–2.95)	0.12 (.08–.18)	0.38 (.29–.48)	0.25 (.19–.34)	172.5
60–72	2.08 (1.74–2.48)	0.07 (.04–.13)	0.21 (.14–.30)	0.14 (.09–.21)	155.5
Total	4.40 (4.09–4.68)	0.20 (.17–.23)	0.34 (.30–.38)	0.23 (.20–.26)	914.4

Source: Kosek et al [41].

Data are presented as incidence rate per child-year (95% confidence interval).

The same study location served as the Peru site of the MAL-ED study and followed 303 subjects <2 years of age from January 2010 to March 2014, a total of 479.1 person-years of surveillance [48, 49]. The study again documented high incidence rates of shigellosis, this time diagnosed by quantitative PCR diagnostics (Table 4). The slight decrease in diarrheal incidence is likely due to a change in surveillance from thrice weekly to twice weekly, which can diminish the detection of mild incident cases. *Shigella* strains from this study (N = 255) were speciated but not serotyped. However, 70.2% of isolates were *S flexneri*, 19.6% were *S sonnei*, and 8.2% were *Shigella boydii*, suggesting that dominant *Shigella* species were stable over the last 2 decades in this population.

**Table 4.** Age-Specific Incidence Rates per Child-Year of All-Cause and *Shigella*-Positive Diarrhea (Diagnosed With Quantitative Polymerase Chain Reaction) by Episode Type in the Peru MAL-ED Cohort

Age, mo	Diarrhea, Any Etiology	*Shigella* spp Positive				Child-Years
		All Diarrhea	Mild Diarrhea	MSD	Dysentery	
0–5	4.96 (4.60–5.35)	0.05 (.02–.11)	0.02 (.01–.07)	0.03 (.01–.08)	0.00 (.00–.00)	133.8
6–11	7.00 (6.55–7.48)	0.37 (.27–.49)	0.26 (.19–.37)	0.10 (.06–.18)	0.02 (.01–.07)	125.4
12–23	5.49 (5.18–5.80)	1.20 (1.06–1.35)	0.85 (.73–.98)	0.35 (.28–.44)	0.11 (.07–.16)	219.8
All	5.74 (5.53–5.95)	0.66 (.59–.74)	0.46 (.41–.53)	0.20 (.16–.24)	0.06 (.04–.08)	479.1

Source: Platts-Mills et al [48].

Data are presented as incidence rate per child-year (95% confidence interval). Severity defined by the Community Diarrhea (CODA) score with 0–4 categorized as mild and ≥5 being characterized as MSD [50].

Abbreviation: MAL-ED, Etiology, Risk Factors and Interactions of Enteric Infections and Malnutrition and the Consequences for Child Health and Development; MSD, moderate to severe diarrhea.

Taken together, these findings demonstrate stable, high rates of diarrhea and shigellosis in the study area over a 20-year period and a consistent and collaborative research network with the capacity to link community-based surveillance with a research laboratory and center of data management and analysis to produce and publish results to inform policy.

## TRAINING AND CAPACITY BUILDING

Iquitos, Peru, has been a center for emerging infectious diseases and global health research for several decades. However, opportunities for leadership in research were not emphasized and projects were generally led by agencies and investigators from the United States and Europe or from research universities based in Lima. EFGH Peru has involved graduate students and local investigators in key positions, such as a co–principal investigator, laboratory lead, and data management leads. Dedicated time to the development of local researchers as independent scientists is supported through a training grant sponsored by the Fogarty International Center, and participation in this and other projects aims to produce investigators as well as high-quality data for the EFGH research consortium. Implementation of inclusive policies in leadership will strengthen the research and possible future vaccine trials as well as transfer of any additional key findings (such as antimicrobial resistance) into regional and national practice guidelines.

## Notes

***Ethics statement.*** This project was approved by the Ethics Review Committee of Asociación Benéfica Prisma and the Institutional Review Board of the University of Virginia. Approval to conduct the study was also obtained from the Regional Government of Loreto.

***Financial support*.** This research was supported by the Bill & Melinda Gates Foundation (grant numbers INV-016650, INV-031791, INV-028721, INV-041730) and the US National Institutes of Health (grant numbers D43TW010913 to M. N. K. and M. P. O., K43TW012298 to F. S., and 1K01AI168493-01A1 to J. M. C.; and T. F. receives support from T32AI055432).

***Supplement sponsorship.*** This article appears as part of the supplement “Enterics for Global Health (EFGH) *Shigella* Surveillance Study-Rationale and Methods,” sponsored by the Bill & Melinda Gates Foundation.
